# Ethical Guidance in Human Paleogenomics: New and Ongoing Perspectives

**DOI:** 10.1146/annurev-genom-120621-090239

**Published:** 2022-05-10

**Authors:** Raquel E. Fleskes, Alyssa C. Bader, Krystal S. Tsosie, Jennifer K. Wagner, Katrina G. Claw, Nanibaa’ A. Garrison

**Affiliations:** 1 Department of Anthropology, University of Connecticut, Storrs, Connecticut, USA; 2 Department of Anthropology, University of Colorado Boulder, Boulder, Colorado, USA; 3 Sealaska Heritage Institute, Juneau, Alaska, USA; 4 Native BioData Consortium, Eagle Butte, South Dakota, USA; 5 College of Arts and Sciences, Vanderbilt University, Nashville, Tennessee, USA; 6 School of Engineering Design, Technology, and Professional Programs; Institute for Computational and Data Sciences; Department of Biomedical Engineering; and Rock Ethics Institute, Pennsylvania State University, University Park, Pennsylvania, USA; 7 Division of Biomedical Informatics and Personalized Medicine, University of Colorado Anschutz Medical Campus, Aurora, Colorado, USA; 8 Institute for Society and Genetics, Institute for Precision Health, and Division of General Internal Medicine and Health Services Research, University of California, Los Angeles, California, USA

**Keywords:** ethics, paleogenomics, ancient DNA, consent, Indigenous, stakeholders

## Abstract

Over the past two decades, the study of ancient genomes from Ancestral humans, or human paleogenomic research, has expanded rapidly in both scale and scope. Ethical discourse has subsequently emerged to address issues of social responsibility and scientific robusticity in conducting research. Here, we highlight and contextualize the primary sources of professional ethical guidance aimed at paleogenomic researchers. We describe the tension among existing guidelines, while addressing core issues such as consent, destructive research methods, and data access and management. Currently, there is a dissonance between guidelines that focus on scientific outcomes and those that hold scientists accountable to stakeholder communities, such as descendants. Thus, we provide additional tools to navigate the complexities of ancient DNA research while centering engagement with stakeholder communities in the scientific process.

## INTRODUCTION

There cannot be a single standard when it comes to the ethics of anthropological research, or even of aDNA research in anthropology. Because aDNA research generally falls outside the domain of institutional review boards, we must regulate ourselves, both through adhering to our field’s sometimes contradictory ethical standards as best we can, and through serious case-by-case consideration and discussion among ourselves, our colleagues within and outside of anthropology, and other interested parties (stakeholders).

—Kaestle & Horsburgh (64, p. 109)

Discussions of ethics in ancient DNA (aDNA) research on human populations has been ongoing since the turn of the century (64, 90, 118). In contrast to research on contemporary human subjects, consent takes on new meaning when applied to genomic samples from Ancestors, who are excluded from biomedical ethics regulatory oversight (125). Here, we use the terms Ancestors and Ancestral to respectfully denote all past human populations, whose physical remains are sampled for paleogenomic research. In the nearly 20 years since the publication of early ethical guidance in paleogenomics, the core ethical challenge of respectfully conducting research on Ancestral individuals has remained under discussion. As O’Rourke et al. (90) foreshadowed in 2000, the ethical, legal, and social concerns within human paleogenomics are “likely to become more, rather than less, important or complicated” (p. 223).

With the advancement of next-generation sequencing technology, there has been a dramatic proliferation of research on ancient genomes from Ancestral humans, from none published before 2009 to more than 1,000 by 2017 (75). This rapid expansion of paleogenomic research highlights concerns regarding data sovereignty and research harms. These issues are intertwined with core questions of who can, or should, give permission for paleogenomic research on behalf of Ancestral individuals. Many discussions within the scientific community are dominated by concerns about paleogenomic sample hoarding (21, 73), funding (52), and the need for more deliberate conservation of the physical remains of Ancestral individuals (43). By contrast, stakeholders such as direct descendants, groups who could be impacted by the research, and the paleogenomic research community (9, 40, 101) have drawn attention to the need for community-based and collaborative research practices to address the potential risks to and concerns of stakeholder communities (10, 111).

Guidance addressing the specific ethical concerns in Indigenous North American contexts (10, 29), African contexts (51, 91), and museums (8), as well as from the standpoints of archaeologists and paleogenomicists (1, 99), have been published by independent researchers. Similarly, professional organizations such as the Society for American Archaeology (102) and American Society of Human Genetics (125) have issued their own frameworks (Tables 1 and 2). However, no guidance has been codified into law or widely integrated into practice. In this review, we provide an overview of the historical and contemporary developments in aDNA ethics by examining the existing published guidance. We then synthesize intersecting themes in various guidelines and respond to emerging issues.

## CONTEXTUALIZING ANCIENT DNA ETHICS

It is a common trope across disciplines that ever-changing technological advances often outpace the development of ethical guidance and legal frameworks (38, 77, 87). However, many ethical concerns related to human paleogenomic research actually predate the genomic era (46, 57). Innovations such as next-generation sequencing and extraction techniques have increased the rate and overall number of ancient genomes sequenced, as well as broadening the scope of research questions in which these methodologies are applied. These technological advancements should not preclude sustained concerns of descendant communities about the procurement of DNA from their Ancestors’ genomes. The legal mechanisms to protect human subjects in biomedical research and repatriate Ancestors in archaeological contexts have been critiqued as inadequate to advance ethical guidance in aDNA research (74). Thus, researchers working in paleogenomics and adjacent fields have drawn from broader, socially responsive theoretical and methodological frameworks to develop best practices for human paleogenomic research.

## Increasing Ethical Awareness for Human DNA Research

Research using biological samples from living humans is subject to research regulations that have been developed in response to grievous violations of human rights. For example, the Nuremberg Code (109) and Declaration of Helsinki (131) were drafted after the human medical experimentation performed by Nazi doctors during World War II came to light (31). These foundational medical ethics guidelines emphasize the rights of research subjects to provide, or withhold, informed consent (12). In the United States, these principles were codified into regulations for research involving human subjects in the Belmont Report (82) after medical research abuses, such as the Tuskegee Syphilis Experiment, continued (18, 63). The Common Rule [Protection of Human Subjects, 45 C.F.R. 46, subparts A–E (2018)], stemming from the Belmont Report, forms the backbone of institutional review board policies and training modules on the responsible conduct of research in the United States. These regulations for biomedical and behavioral research using human subjects center the rights of research participants over those of researchers and broader society through the core principles of respect for persons, beneficence, and justice (24). The underlying principles of the Common Rule in the United States have also been embraced internationally [e.g., by the World Health Organization, the Council for International Organizations of Medical Sciences (33), and the World Medical Association (131)] and are generally regarded as being foundational to the international human right to science [120; 121, article 27(2); 123, article 15(b)].

Despite these existing research guidelines, human genetics (and later genomics) research has also entailed ethical missteps. Well-documented case studies such as the Havasupai Tribe’s lawsuit against the Arizona Board of Regents over misuse of DNA samples (58) (see the sidebar titled Havasupai Tribe Versus the Arizona Board of Regents) and genetic diversity projects such as the Genographic Project (see the sidebar titled The Genographic Project) reflect the unequal power dynamics that frequently exist between researchers and research participants. Additionally, the cases demonstrate how researchers might lack the cultural competence to adequately anticipate potential harms to research participants. These ethical oversights demonstrate that broad, institution-level regulations such as institutional review boards can provide baseline expectations for researchers; however, significant gaps in research guidance remain. Thus, on the ground, it frequently falls to individual researchers to anticipate and navigate the various ethical challenges, which differ from project to project (64, 90, 118).

## Diverging Ethical Standards for Nonliving Human Ancestors

In the field of human paleogenomic research, with its intimate ties to human genomics and archaeology, these ethical concerns are compounded. Despite engaging with human biological samples, paleogenomic research is currently not subject to the same regulatory frameworks as research using genomic samples from human subjects, as Ancestors are considered “nonliving” individuals. From this perspective, ancient, or nonliving, Ancestors cannot give or withhold consent. While institutional review boards could impose constraints on researchers that go beyond the regulatory requirements of the Common Rule, there are no known examples of human aDNA studies being conducted pursuant to such oversight. Thus, although paleogenomic researchers use human biological samples, they are not held accountable to the same regulations as researchers working with living human subjects. Additionally, there is a legitimate question as to whether a biomedical ethics framework—with its heavy emphasis on autonomy—is appropriate to guide responsible aDNA research practices. Importantly, these biomedical frameworks reflect a narrow, Euro-centric worldview. Alternative ethical frameworks, such as environmental ethics, public health ethics, forensic ethics, and yet-to-be-designed ethical frameworks (26, 50, 65, 69, 104, 129), might offer perspectives that emphasize other values relevant to paleogenomic research (e.g., solidarity and interrelatedness). While some regulatory frameworks do address nonliving individuals, such as the Native American Graves Protection and Repatriation Act (NAGPRA) [25 U.S.C. §§ 3001 (1990)] in the United States, this legislation does not address whether or how aDNA research can be done lawfully, as the statute is silent on the topic of research. NAGPRA focuses specifically on the process of repatriating human remains, and once that process has been completed, claimants have legal control over whether the remains may be used in scientific research. Additionally, NAGPRA is not applicable to one of the flagship museum collections of the United States, the Smithsonian Institution (including both the National Museum of the American Indian and National Museum of Natural History), which is governed by the National Museum of the American Indian Act [Pub. L. 101-185, 103. Stat. 1336 (1989), as codified at 20 U.S.C. §80q] as amended by the National Museum of the American Indian Act Amendments of 1996 [Pub. L. 104-278, 110 Stat. 3355 (1996)] (83, 85).

The broader discourse that was spurred in archaeology and beyond around issues such as repatriation (19, 36, 119), identifying descendant communities (93), and potential impacts of archaeological findings on living communities (127) highlights analogous concerns in paleogenomic research ethics. Some initial discussions on professional research ethics in human paleogenomics intersect with these issues. In 2002, Kaestle & Horsburgh (64) published one of the earliest sets of guidance regarding paleogenomic research ethics. They urged researchers to consider the methodological risks of the proposed aDNA extraction (such as whether a sample is likely to yield aDNA or if less destructive methods might be more suitable), as well as to seek permission from and evaluate impacts on stakeholders beyond the scientific community (64). Guidance from Sealy (97) in 2003 spoke to a more global context of archaeological research with Ancestral individuals and explicitly highlighted the need to respect both the wishes of local communities and the scientific research value of Ancestors’ biological remains. Both of these early frameworks illustrated emerging tensions in balancing concerns about scientific outcomes with the concerns of other stakeholders, such as descendants. However, unlike biomedical research guidance—which explicitly states that research participants’ rights should not be overshadowed by perceived societal benefits from research outcomes—these early examples of paleogenomic research ethics provided no such direction about how to prioritize potentially competing interests.

Sealy (97) also advocated for respecting the scientific value of the physical remains of hominid Ancestors, in line with the more global framing of their discussion. In 2008, Hublin et al. (59) provided guidelines specifically for research with the biological remains of hominids. As paleogenomic research in these early phases was considered to be high risk due to its low DNA recovery rates and high susceptibility to contamination, these guidelines were concerned exclusively with the scientific merit of destructive sampling of hominid remains. The remains of these individuals were positioned as valuable scientific commodities, and thus the ethical concerns were oriented around minimizing the potential for unsuccessful destructive sampling and, in turn, minimizing perceived harms to scientific stakeholders regarding “wasting” valuable scientific samples. The concerns of descendants and other affiliated communities regarding destruction of and research on Ancestors’ remains were given little consideration. Instead, members of the research community positioned themselves as the primary stakeholders and decision makers regarding research ethics, risks, and benefits.

In the years since the publication of these initial guidelines, concerns regarding the research ethics and implications of aDNA studies have continued to emerge. Indigenous communities and scholars have been on the forefront of highlighting harms from paleogenomic research that have yet to be adequately addressed. In one recent example, paleogenomic research by Kennett et al. (66) involved Indigenous Ancestors removed from Pueblo Bonito in Chaco Canyon. These Ancestors were categorized as culturally unidentifiable by the American Museum of Natural History, which then retained legal control of the Ancestors’ remains and allowed biological samples to be collected from 14 Ancestors for aDNA analysis and radiocarbon dating (32). The researchers who accepted these samples deferred to the museum and did not independently consult with or seek permission from any Indigenous nations prior to collecting the samples or beginning research (29, 32). In response, Claw et al. (29) highlighted best practices for research with Indigenous Ancestors who have been classified as culturally unidentifiable through NAGPRA. Recommendations include consulting broadly with tribes who not only have been culturally affiliated with the Ancestors, but also have historical and geographic ties to the lands where Ancestors have been laid to rest. The ways in which NAGPRA severs ties between Ancestors who have been categorized as culturally unidentified and their cultural and biological descendant communities provide another example of why relying on current regulatory frameworks is inadequate for defining ethical research practices.

As aDNA research proliferates through enhancements in research technology and capacity (75, 89, 100), more sophisticated research frameworks have emerged to grapple with enduring ethical challenges. Increasingly, these guidelines address issues of long-term data and biological sample stewardship. Still, there remain embedded tensions in how these guidelines position stakeholders to make decisions regarding appropriate research directions, acceptable risk, and how research outcomes and products are managed in the long term. Guidelines from Sirak & Sedig (99), Austin et al. (8), and Alpaslan-Roodenberg et al. (1) address the debate in genomics about data and biological sample management. However, these frameworks continue to position paleogenomic researchers as the arbitrators of whether and how paleogenomic research should be conducted. While these researchers encourage “engaging” (1) or “consulting” (8) with stakeholders beyond the research community, they do not cede the power to make final decisions about the research. They thus privilege the agency of scientists, the main beneficiaries of research, over other stakeholders who face disproportionate risks from the results of paleogenomic research (111).

The frameworks provided by Sirak & Sedig (99), Austin et al. (8), and Alpaslan-Roodenberg et al. (1) point to national policies and regulations as mechanisms for ensuring ethical research conduct. Compliance with the law is necessary but is not sufficient to define responsible conduct of aDNA research. Few would disagree with the recommendation that researchers should comply with all applicable laws and regulations; however, existing guidance typically fails to provide adequate counsel regarding how researchers could or should resolve conflicting obligations. Public and private policies from different levels (e.g., local, state, federal, tribal, and/or regional governments or institutional units) or locales (e.g., where Ancestors’ remains are located, where research is performed, and/or where relatives or descendants are living) might be incompatible. Laws and regulations can also change over time, and those changes might not coincide neatly with project deadlines or phases of research. Existing regulations might not adequately protect unique concerns of marginalized communities with respect to research. The oft-quoted phrase that the law is “marching with medicine but in the rear and limping a little” (79, p. 395) remains appropriate in the context of paleogenomic research ethics.

By contrast, guiding questions from Bardill et al. (10) and recommendations from Wagner et al. (125) attempted to recenter stakeholders beyond paleogenomicists in the research process. These frameworks call for researchers to directly address the concerns of stakeholder communities and include them as active decision-making partners in the research process. In doing so, they intersect with broader discourses and theoretical trends in archaeology that advocate for sharing agency over the research process between scientists and other stakeholders. Community-based archaeology (6, 7) urges researchers to equitably engage stakeholder communities as research partners, similar to the principles of community-based participatory research used in human biomedical and behavioral research (61). More specifically, Black feminist (11, 44) and Indigenous (5, 86, 126) archaeology methods reconcile exploitive archaeological research practices that built narratives of Black and Indigenous histories while excluding members of these communities from the field. Thus, these methodological and theoretical frameworks center descendant community knowledge and values while holding researchers accountable to the academically marginalized communities represented in their research.

Taken as a whole, the most recent body of ethical guidance addressing paleogenomic research highlights where tensions remain in navigating diverse, and sometimes competing, stakeholder concerns. These frameworks attempt to address enduring and emerging ethical considerations. While some guidelines intentionally reframe paleogenomic research methods to engage the concerns of stakeholders beyond the scientific community, others emphasize the need for conducting robust science even if this research conflicts with the concerns of stakeholders such as descendant communities.

## SYNTHESIZING ANCIENT DNA GUIDELINES

As of 2021, more than 11 sets of guidelines on ethics in aDNA research have been published by independent researchers, along with 2 sponsored by professional organizations (Tables 1 and 2). Despite growing ethical discussions within and alongside human paleogenomics over the past 20 years, field practitioners have yet to agree on a set of standardized guidelines. This speaks to the complexity of the ethical considerations encompassed within aDNA research; it is likely that no singular framework can adequately address the evolving concerns of diverse stakeholders (64, 90, 118). Having introduced and contextualized the primary ethical frameworks currently guiding human paleogenomic research, we now highlight key issues facing the field today. Many of these challenges were identified in initial frameworks guiding paleogenomic research but evolved and endured as the field expanded. As we synthesize current guidance on these key topics and discuss intersecting knowledge from fields beyond paleogenomics, we pay special attention to how various stakeholders are positioned and prioritized. In doing so, we highlight the gaps to overcome in order to adequately guide paleogenomics.

### Identifying Descendant Communities and Community Stakeholders

Conceptualizing who is a descendant in relation to Ancestral individuals is complex. Community, legal, and institutional understandings of who is categorized as a descendant can vary locally and globally and might or might not privilege biological heredity over other forms of relatedness. Definitions of descent might also encapsulate broader cultural or geographical relationships between persons and communities connected to Ancestors. Identifying these descendant relationships is critical, despite the challenges, as guidelines for genomic research often point to descendant communities as among the primary stakeholders to be consulted for consent to conduct genomic research involving Ancestors. Importantly, descendant communities might also embody feelings of personal responsibility to Ancestors, as well as bear disproportionate risk of harms stemming from paleogenomic research on Ancestors.

In the context of Indigenous Ancestors in the United States, NAGPRA outlines various characterizations of descendants, including lineal descendants, culturally affiliated communities, and descendant communities. A lineal descendant is an individual who can trace their ancestry or lineage directly to an Ancestral individual (103). NAGPRA also uses the term culturally affiliated when referring to a relationship of shared group identity that might be reasonably traced historically or prehistorically between a present-day tribe and an identifiable earlier group [43 C.F.R. § 10.14 (1995)]. Cultural affiliation is determined by using the totality of available evidence based on geography, kinship, biology, archaeology, anthropology, linguistics, folklore, oral tradition, history, and other relevant information or expert opinion [43 C.F.R. § 10.14 (1995)]. Other definitions of Indigenous descendant communities refer to geographic proximity between modern tribal communities and the locations of Ancestors’ remains or to the original inhabitants of the land where Ancestors were found. For example, the National Park Service defines traditionally associated communities as those having no biological connection to an Ancestor but nonetheless having close ties to places where Ancestors have been found (84). In terms of repatriation, NAGPRA prioritizes lineal descendants, then modern tribal communities of the land where Ancestors were found, then the closest culturally affiliated tribes, and finally the aboriginal inhabitants of the land where Ancestors were found [43 C.F.R. § 10 (1995)].

Conceptions of who constitutes a descendant community in the United States also vary outside of Indigenous contexts. Descendant communities might be difficult to determine because of historical policies of forced removal, enslavement, and assimilation. In particular, for African American communities for whom “centuries of displacement and sparse genealogical records…[make it] difficult to link a set of human remains to specific Black descendants” (37, p. 339), identifying descendant communities can be challenging (37, 70). In 2020, the Senate unanimously passed the African American Burial Grounds Study Act to “conduct a study of ways to identify, interpret, preserve, and record unmarked, previously abandoned, underserved, or other burial grounds relating to the historic African American experience” [S.2827, 116th Cong. (2020), section 3(a)], and there is current advocacy for the enactment of an African American Graves Protection and Repatriation Act (37), similar to NAGPRA.

The curation history of Ancestors housed in museums can further complicate the identification of appropriate descendant or stakeholder communities. As many North American and European natural history and archaeology museums are intertwined with global colonial empire-building enterprises, Ancestors housed in these collections might be geographically disconnected from descendant communities (39). Thus, designations of culturally unaffiliated Ancestral individuals within museums might not reflect the current knowledge of community descendants. There are also potential conflicts of interest, as museums were often the drivers of the collection of Ancestral individuals and now have the final say on what happens to those individuals (32).

Recent ethics guidance for paleogenomic research has recommended engaging key stakeholders as part of the research process (Table 2). Specifically, four of the published sets of guidelines mention engaging with descendant or culturally affiliated communities (8, 10, 29, 125). The question of whom to consult and how to carry out the consultation is most challenging when descendant communities are difficult to define. Claw et al. (29) and Wagner et al. (125) provided additional guidance for consultation in situations where there are no direct or obvious descendant communities. In Indigenous North American contexts, Claw et al. (29) suggested that tribal experts should be consulted, especially with tribes who have historical and geographic ties to the areas where Ancestors were interred. Wagner et al. (125) recommended first consulting with regional governing bodies where the Ancestors were recovered or are currently housed, or consulting with other researchers or curators who are supervising the Ancestors or might have worked with similar communities.

Alpaslan-Roodenberg et al. (1) suggested that using frameworks developed in Indigenous North American contexts to guide consultation and engagement is actually harmful in a variety of situations worldwide where there is no clear descendant community. To support their claim, they cited examples where the concept of Indigeneity creates conflict or contributes to xenophobia and nationalist narratives. Instead, they recommended abiding by legal regulations from institutional, local, or national bodies. However, the authors failed to grapple with the possibility that these governing bodies can have entrenched histories with colonialist institutions, or can be colonialist institutions themselves (15, 117). The individuals they engage with might be strategically selected to legitimatize the governing bodies’ decisions, thereby excluding other advocates of Indigenous or local community interests from the discussion. Thus, it is important for researchers to critically interrogate the role of these institutions, address potential power imbalances, and consider how communities might be disenfranchised by them.

Ultimately, the existing guidelines serve as a starting point for researchers to think critically about the communities that might be connected to the Ancestors in question or affected by the outcomes of paleogenomic studies. Given the various approaches to defining descendant communities, as well as the complex shared lineages and relationships among many different communities, it is not surprising that conflicts arise. However, identifying descendant communities and community stakeholders is necessary to ensure that proper consent is obtained. Furthermore, seeking permission from descendant communities before conducting any aDNA research is important to establish trusting relationships that support the rights of communities to be involved in research. Ideally, memorandums of understanding or other formal agreements should be drafted together and signed by all interested parties. Evidence of permission should be submitted with manuscripts, stated during presentations, and archived for posterity.

### Engaging Communities in Consent for Ancient DNA Research

Traditional notions of consent drawn from biomedical contexts are not equipped to navigate the challenges of human paleogenomic research. The notion of informed consent, as defined in regulatory frameworks such as the Belmont Report [Federal Policy for the Protection of Human Subjects, 82 Fed. Reg. 7149 (2017)] and the Common Rule [Protection of Human Subjects, 45 C.F.R. 46, subparts A–E (2018)], is not easily translatable to work with nonliving Ancestors. The concept of consent for human paleogenomic research was introduced by Kaestle & Horsburgh (64) through a discussion of proxy consent. The authors noted the challenges inherent in determining who is best positioned to evaluate the appropriateness of extracting aDNA from Ancestors. Moreover, discussions of consent in human paleogenomic research have moved beyond simply obtaining permission to collect and/or analyze samples from Ancestors (125).

Critically, the issue of who should serve as proxies to consent on the behalf of Ancestors is an ongoing point of discussion within the field. Stakeholders such as museum curators and collections managers, archaeologists, genomicists, and descendants have all been positioned as proxies for Ancestors in paleogenomic research (8, 10, 59, 91, 99). Sirak & Sedig (99), as well as Austin et al. (8), emphasized the role of museums, archaeologists, and genomicists in curating and granting access to many Ancestors’ remains for paleogenomic research. For example, Sirak & Sedig (99) specifically highlighted the importance of collaborative project planning and decision-making between archaeologists and paleogenomicists, including which Ancestors should be sampled and for what research questions. Considering consent for research from this perspective, these authors were concerned with mitigating potential harm to the Ancestors by ensuring the physical conservation of their biological remains, as well as the overall feasibility and scientific impact of the proposed research.

Descendant communities have also been empowered to serve as proxies for Ancestors in research. This recognizes the interrelationship between Ancestors and descendant and other closely affiliated communities, including how paleogenomic research on Ancestors can both harm and benefit descendant communities (96). Because of this interrelationship, consent from descendant communities may be a more dynamic process that shifts from obtaining permission to sustained consultation and collaboration. Bardill et al. (10), Claw et al. (29), and Wagner et al. (125) all highlighted the many stages throughout the research process when key community leaders and stakeholders should be engaged to make decisions. Beyond access to Ancestors’ remains and biological samples, consultation may also address the use of existing genomic data from Ancestors, interpreting and communicating research results, and the long-term custodianship of Ancestors’ biological materials and data.

Involving descendant communities in aDNA research can avoid creating, or mitigate, potential harms for these stakeholders, such as exacerbating distrust of researchers, increasing psychological harms resulting from disturbing Ancestors, or conducting research that goes against community interests. For example, Claw et al.’s (29) discussion of paleogenomic research on Ancestors from Chaco Canyon illustrated that consulting and collaborating with descendant communities would have avoided inflicting harms. Researchers used disrespectful, dehumanizing language to reference Ancestors in the scientific publication and ignored community-held knowledge about the Ancestors and site history (66).

Coauthoring publications and other means of communicating research outcomes is another opportunity for researchers to ensure consent from descendant and other affiliated communities and prevent them from being inadvertently harmed by research findings. Stakeholders should be given opportunities to request redactions or technical corrections prior to a manuscript’s submission for publication. For example, the Navajo Nation Human Research Review Board reviews manuscripts for “technical content and validity, organization of content, readability, as well as assurance that they are consistent with the goals, intent and policies of [the] Code” [Navajo Nation Human Research Code, Navajo Nation Council Resolution CO-106-95 § 3269 (2002)]. This is similar to industry partners reviewing manuscripts to ensure that no disclosure of proprietary information occurs either intentionally or inadvertently and that appropriate acknowledgments of contributions or authorship have been made (22).

Alpaslan-Roodenberg et al. (1) agreed that engagement with diverse stakeholders is important, yet they argued that “researchers cannot ethically participate in a study” if stakeholders beyond the research team review manuscripts prior to their publication (p. 44). While research must be shielded from inappropriate influences that undermine scientific integrity, such as financial and nonfinancial conflicts of interest and censorship (95), genomic research collaborations often involve and benefit from partnerships in which stakeholders beyond the authorship team have an opportunity to review proposed manuscripts. To suggest that prepublication review is absolutely unethical or antithetical to science is a gross oversimplification, is indicative of limited moral imagination (116), and does not respect community input into collaborative research. There are already examples of how paleogenomicists and researchers have worked together on study design and the publication of research results (9, 40).

Frequently, paleogenomic research also compares aDNA from Ancestors with DNA from living descendants. These studies might analyze population histories and migration, examine signatures of biological evolution and selection, and identify genetic relationships for the purpose of repatriation (25, 71, 78). In one recent example, paleogenomicists examined the genetic relationship between Lakota Sioux leader Tatanka Iyotake, also known as Sitting Bull, and great-grandson Ernie LaPointe (78). Tatanka Iyotake is best known for defeating General Custer at the Battle of Little Big Horn. Killed in 1890, he was initially buried in North Dakota. Later, his remains were reportedly moved to South Dakota. LaPointe has sought rights to exhume this latter gravesite and Tatanka Iyotake’s remains (34). LaPointe and 13 unrelated Sioux individuals gave oral and written consent to include samples of their DNA in a study that yielded genetic evidence to support LaPointe’s claim of lineal descent from Tatanka Iyotake (78). The study, however, raises several ethical concerns regarding consent and engagement [see the sidebar titled Identifying Relatives of Tatanka Iyotake (Sitting Bull)].

In another example, colonial settlers and anthropologists in Australia collected biological materials such as hair, blood, and other bodily remains from Indigenous peoples (92). Some of these samples have subsequently been analyzed in aDNA research (107). In recent years, efforts to repatriate these culturally meaningful legacy collections have been made by Australian Aboriginal and Torres Strait Islander peoples. Another related effort is the engagement of Elders across Australia who donated DNA to trace and track down remains of Ancestors held in museum collections (132). In this study, DNA from living Indigenous individuals in Australia was compared with ancient nuclear and mitochondrial genomes to link and repatriate Ancestral remains to descendant communities. Elders were involved in the process and offered guidance for engagement with communities, provided oral histories and contexts for samples and repatriation, and were authors of the resulting publication (92).

There are a variety of ways that consent can be conceptualized and utilized in human paleogenomic research. At a minimum, aDNA researchers should identify key stakeholders from descendant or closely affiliated communities and engage them in focused discussions about research intentions, with the goal of reaching a formal agreement on how the research will proceed (10, 91). In this context, consent should be formally documented and clearly outline agreements regarding the goals of the research, plans for protection of data and confidentiality, considerations for data collection, public use of community and group identifiers, whether any community-level approvals have been granted, and strategies to minimize risks to participants and affiliated communities. Paleogenomicists may consider applying frameworks such as dynamic consent, which emphasizes the ever-evolving nature of research by requiring researchers to remain in dialogue with participants throughout a study (20). Good-faith community engagement also requires recognition by paleogenomicists that consent for research can be withheld or withdrawn, despite scientific interest in a topic. Paleogenomicists must not overstep by presuming that aDNA research involving Ancestors and descendants should be done (14). This intersects with broader discussions of Indigenous sovereignty in relation to genetic research (49). In the United States, tribal communities and Indigenous bioethicists have urged researchers to uphold Indigenous sovereignty in consenting to and guiding research with Ancestors (113). Similarly, the United Nations Declaration on the Rights of Indigenous Peoples emphasizes the rights of Indigenous peoples globally to govern human and genetic resources (122). Despite the challenges of translating and applying the biomedical concept of informed consent to research with Ancestors, engaging descendant communities and other stakeholders is a critical component of conducting paleogenomic research.

### Scientific Feasibility

Ethical guidance in paleogenomics is heavily focused on scientific feasibility, or the ability of the researchers to (*a*) answer questions of anthropological or scientific importance with a realistic research plan, (*b*) choose DNA sampling to minimize damage, and (*c*) carry out the research plan. However, orienting ethical guidance around considerations of scientific feasibility privileges the values of the scientific community. These implications must be critically assessed, as scientists have repeatedly caused harm to marginalized stakeholder communities (41, 45, 54, 88). Here, we discuss how scientific feasibility intersects with other ethical considerations in aDNA research.

Much of the proposed ethical guidance in paleogenomics highlights the need to minimize unnecessary or unwarranted damage to Ancestors’ physical remains (1, 59, 64, 91, 99). Analysis of Ancestors’ remains provides unique insight into evolutionary and archaeological research questions. This underlies the anxiety among members of the field that destructive testing should not be undertaken without in-depth consideration of the scientific ramifications of the proposed research (8). There are inherent methodological challenges in aDNA research: The population of Ancestors potentially accessible for aDNA research is finite, DNA preservation is not guaranteed, and aDNA extraction is a destructive process (80, 99). Minimally invasive approaches to aDNA extraction have been developed, whereby skeletal elements can be soaked in an ethylene-diaminetetraacetic acid (EDTA)–based buffer to extract DNA instead of drilling and removing bone from the Ancestors, reducing physical destruction of their remains (16, 56). Despite these innovations, scientists remain concerned that any depletion of Ancestors’ remains through aDNA research, which is perceived as a “risk” (72), must be outweighed by the newly generated scientific knowledge (i.e., “benefit”).

In early comments addressing aDNA research ethics, Kaestle & Horsburgh (64) stated that “destructive analysis should only be undertaken in cases where the results are likely to inform important debates or provide data to test interesting hypotheses” (p. 106). This view is similarly reflected in human evolutionary studies, where Hublin et al. (59) wrote that the “scientific question addressed should be important enough to justify invasive sampling of hominid remains and should not be answerable by other means” (pp. 756–57). Most recently, Alpaslan-Roodenberg et al. (1) focused three of their five proposed guidelines on the scientific research process: preparing a detailed research plan, minimizing damage, and ensuring that data are made available after publication. This is similar to guidance previously contributed by Sirak & Sedig (99), which was directed toward building shared research frameworks between archeologists and paleogeneticists to enhance collaborative approaches to understanding human history.

When ethical aDNA guidance is written by geneticists and archaeologists for other geneticists and archaeologists (1, 8, 99), it puts goals of the research community over those of other stakeholders, such as descendants or community members. While researchers are stakeholders in the research process, they should not exclusively evaluate notions of acceptable use of Ancestors’ remains. Likewise, understanding of research harm in paleogenomics cannot be limited to minimizing damage from destructive sampling of Ancestors’ remains. Harms can also affect living people and communities.

Existing ethical guidelines also call for researchers to evaluate their capacity to successfully carry out the proposed paleogenomic research (1, 59, 64, 91, 99). These research guidelines focus on assessing the capacity of researchers’ laboratory-based methodological skills, particularly in techniques that minimize the destruction of the Ancestors’ physical remains (1, 59, 91, 99). They do not address the need for researchers to professionally develop skills in community engagement or community-based research, or to enhance research infrastructure and capacity in collaboration with local stakeholder communities.

Ethical guidelines written from the perspective of other, sometimes overlapping stakeholders, such as Indigenous scholars or trained bioethicists, are critical of privileging the potential scientific outcomes of paleogenomic research (10, 29, 125). Guidelines by Bardill et al. (10) and Wagner et al. (125) decenter the scientist by incorporating diverse perspectives directly into aspects of the research process, such as hypothesis building (28). In doing so, they engage the needs and priorities of living relations and stakeholder communities to more holistically assess risks and benefits of the proposed research and mitigate potential harm (60). Harm reduction thus expands outside the scope of laboratory-based methodologies. Scientists have a responsibility to evaluate the merits, potential, and pitfalls of proposed aDNA research before proceeding. However, due diligence should encompass engaging the diverse communities beyond scientists who are also stakeholders to this research. This will ensure the research is responsive to the values and responsibilities of these communities in relation to Ancestral individuals.

There are also unequal distributions in the field of aDNA research in which laboratory facilities, funding, and training reflect the lack of equitable access to technology and other resources (21, 73). As of 2018, there were 75 recorded aDNA laboratories across the globe, with the majority located in Europe and North America (94). Of these laboratories, only one is in South and Central America, four are in Asia, six are in the Indo-Pacific region, and none are in Africa. The majority of large-scale funding initiatives for aDNA projects are available only to laboratories in Europe (52). Such dynamics perpetuate helicopter research in which aDNA samples are extracted by researchers, taken to other countries, and exploited for professional gain without subsequent engagement with local descendant communities (91).

To develop more equitable research practices in human paleogenomics, Wagner et al. (125) specifically highlighted the responsibility of researchers to build capacity with stakeholder communities when conducting aDNA research in ways meaningful to the community members, including educational initiatives, research training, and coauthorship opportunities. Bardill et al. (10) also discussed capacity building, highlighting the Summer internship for INdigenous peoples in Genomics (SING) workshop to train community stakeholders and Indigenous students in the applications and limitations of genomic methods, as well as to broaden bioethical considerations for Indigenous communities (28, 49). Others have called for paleogenomicists to turn their attention inward and build their own capacity to engage in respectful and culturally responsive ways with descendant communities (9). These initiatives are important to cultivate a more equitable future in genomic research (28).

### Assumptions Underlying Open Data Sharing

Open access data are touted as necessary to expand the transparency and accountability of research while also helping to accelerate innovation by the scientific community (17). In particular, sharing paleogenomic data among researchers and institutions has been emphasized as a method of advancing aDNA research (3). Importantly, questions of data access are intertwined with issues of consent and community engagement in human paleogenomic research. Recent ethical guidance has begun to address this topic (1, 8, 99, 125) but does so from contrasting perspectives (Table 2). For instance, Alpaslan-Roodenberg et al. (1) and Sirak & Sedig (99) advocated for the mandatory deposition of sequencing data in open or public repositories for continued access by investigators, claiming that this is necessary to critically reevaluate the accuracy of paleogenomic findings, a perspective mirrored by Austin et al. (8). By contrast, two of the tenets in the guidance from Wagner et al. (125) were to “develop plans to report results and manage data” and “develop plans for long-term responsibility and stewardship” (p. 183). The specific recommendation to “develop plans” highlighted the importance of meaningfully engaging with communities about data management as the main determinant in decision-making, rather than centering scientific and museum stakeholders as the primary decision makers.

Long-term data management has become increasingly important for researchers and stakeholder communities. However, the ethics underlying data sharing relies on several assumptions that must be critically evaluated to ensure that open paleogenomic data do not further entrench power imbalances between researchers and stakeholders who have been historically excluded from research discussions, such as descendant communities.

Data are valuable assets to those who can access them (42) and are often discussed in terms of ownership and stewardship. Equating open data with “democratizing” data for stakeholders (115, p. 184) implies that the value associated with research data can and will be equitably distributed (67). Existing ethical guidelines by paleogenomicists advocating for open data assume that the value inherent to aDNA data can be equally distributed to all stakeholders. Unfortunately, however, DNA data have historically benefited research groups—commercially (42), professionally, or both—to the neglect of descendants’ rights and interests. For example, grant-funded research frequently awards indirect costs to paleogenomicists’ institutions (68). Descendant communities are often not the awardees of these types of grants and thus do not similarly financially benefit from engaging in this research. Whether data value and power can be equally distributed to stakeholders is another key concern. Members of descendant communities might or might not choose (or be asked) to engage in the research process, and data valuation might fundamentally be dissimilar according to different needs and interests.

A second assumption made by proponents of open data is that the benefits of data sharing outweigh the risks. However, those who benefit from open sharing of aDNA data are unilaterally paleogenomicists and stakeholders who are interested in collectivizing or advancing aDNA work. Data sharing could negatively impact historically disempowered peoples and communities (60), who might question the utility of aDNA work involving their contemporary descendants and Ancestors. Disproportionate risks to disempowered peoples include but are not limited to breaches to data privacy (27), stigmatization (112), overbiologization of traits misattributed to “genetic race” (81, 108), and misappropriated ownership of data and extraction or exploitation of data. These risks are unlikely to be experienced by researchers, and discounting them is commensurate with overselling the promises of open data for the benefit of those likely to benefit from the data.

Finally, those advocating for open data in paleogenomics assume that all stakeholders are empowered to make data decisions and that the level of empowerment is commensurate with the benefits and risks of contributing data. We should question open data principles and policies that shift data governance and stewardship toward agents who access and benefit from data in a manner unchecked by communities at risk. Centering paleogenomicists as the arbiters of frameworks for accessing data when they are poised to disproportionately benefit is a conflict of interest (111). Open data policies that center researchers’ “rights” to access data shift power and agency toward researchers and away from descendant communities (60, 114). Therefore, FAIR principles (findability, accessibility, interoperability, and reusability) (130) that center the data access needs and values of researchers grant too much decision-making authority to researchers working outside of community-level governances. Considering CARE principles (collective benefit, authority to control, responsibility, and ethics) (23) in conjunction with FAIR principles for sharing of aDNA data is one way to reconceptualize data stewardship as a responsibility rather than a right of access by researchers. To achieve this, aDNA data should be considered “on loan” (4), with permissions revocable if data are misused (114). To echo the Society for American Archaeology (102), working with human Ancestors should be considered a privilege, not a right.

Some researchers, such as Alpaslan-Roodenberg et al. (1), have pointed to Indigenous-led biological-data repositories as potential venues for supporting the storage and distribution of data for “purposes beyond those covered by the original research agreement” (p. 44). While this approach can support governance by Indigenous peoples, this description improperly depicts the availability of data to be managed by Indigenous institutions and repositories. In fact, Indigenous biological-data repositories are a means of exerting Indigenous data sovereignties (47, 115) through which consent from Indigenous peoples is sought at both the individual and community levels. By these measures, only aDNA research in which Indigenous communities have consented to engage with trusted partners would be permitted. In this sense, Indigenous biological-data repositories implement community-based participatory models, in which Indigenous peoples might be more passively engaged as part of the data generation process, and represent a step toward tribally driven research (76), in which Indigenous peoples and communities are empowered to manage their data commensurate with Indigenous data sovereignties (60, 115).

Collecting data for the mere purposes of building data conglomerates should not be the goal of research. It is critical for both researchers and other stakeholders to question why aDNA data are being collected and who benefits from the data; this must be revisited throughout the research process. This is why broad consenting models that grant data access carte blanche, without reconsenting communities, are inadequate for ascertaining informed consent. Instead, paleogenomicists should consider adaptive research consent models such as dynamic consent (20), which is an ongoing process that continually engages research participants. Community data collectives that place data decision-making authority within the hands of affected communities, not researchers, are another useful model (47, 115). Ultimately, the discussions addressing data sharing in paleogenomic research reflect the evolving challenges of engaging multiple and diverse stakeholders in research with Ancestors.

## THINKING ETHICALLY

Increasingly, ethical frameworks and guidelines in human paleogenomic research emphasize community engagement and collaboration as a means to navigate the complex challenges of issues such as consent, appropriate research methods and questions, and acceptable use and curation of genomic data. Claw et al. (29) noted that studies involving Native American Ancestors should be done in consultation with culturally affiliated tribes and tribes with historical and geographic ties to the human remains. Bardill et al. (10) reiterated this point and further suggested that aDNA researchers should identify the peoples who should be consulted if no descendant or culturally affiliated communities are known. Wagner et al. (125) also emphasized the importance of formal consultations with relevant communities before, during, and after aDNA research and offered a distinct set of key questions for researchers and communities to consider. The question of what constitutes meaningful collaboration with stakeholder communities, however, might not be fully grasped within the paleogenomic research community. Guidance from researchers such as Alpaslan-Roodenberg et al. (1) noted the importance of engaging with stakeholders such as local communities but continued to prioritize the needs and values of the scientific research community. For instance, they stated that it is important to “confront colonial legacies’” by providing training and capacity-building opportunities to help shape research design (1, p. 43). However, this imposes a Euro-centric research framework as a means to “empower” communities, without asking or considering what stakeholder communities themselves might actually consider meaningful engagement (30). This highlights the continuing tensions that hamper the development of a singular set of best practices for research with Ancestors.

**Figure 1:**
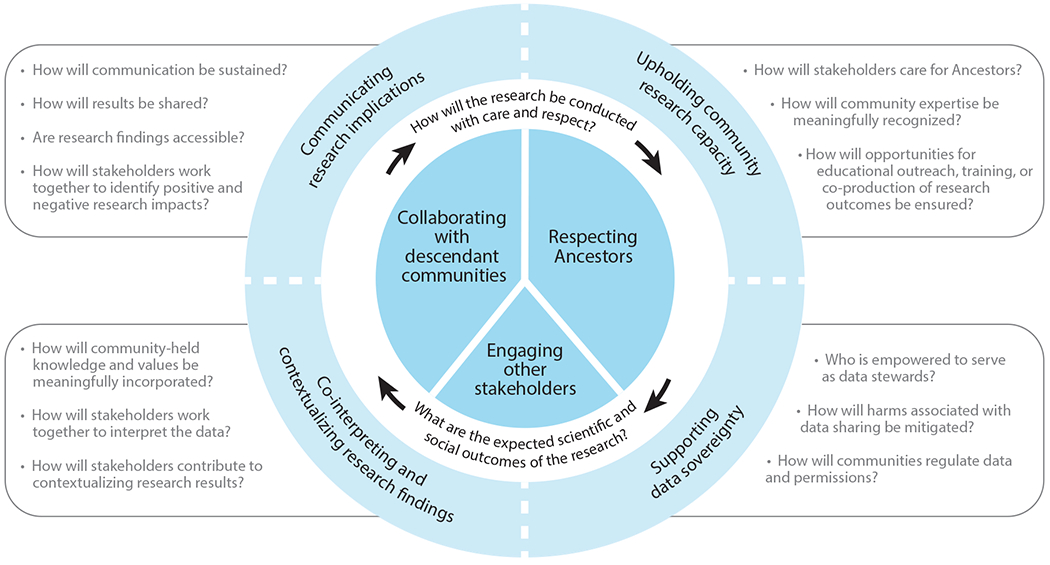
Ethical ethos for paleogenomic research. Three main stakeholder groups—Ancestors, descendant communities, and other stakeholders (i.e., nondescendant communities, researchers, and institutions such as museums)—are positioned as central to the research process, with verbs identifying researchers’ primary relationship to them. The actions “collaborating with descendant communities” and “respecting Ancestors” occupy proportional space to each other but hold greater weight than “engaging other stakeholders” to reflect how researchers might balance the diverse values of these different communities. Two questions at the core guide researchers to think about the ethics of research methods and outcomes. These questions are surrounded by an outer circle containing themes that enhance ethical paleogenomic research practices: upholding community research capacity, supporting data sovereignty, co-interpreting and contextualizing research findings, and communicating research implications. Additional research questions to guide researchers and community stakeholders in obtaining a deeper understanding of these concepts are listed in the adjacent boxes. Together, the figure and accompanying questions encourage researchers to engage each stakeholder community throughout the research process.

Rather than proposing another set of recommendations, this review focuses on cultivating an ethical ethos in paleogenomic research. Recognizing the importance of identifying stakeholder communities, seeking permission, and considering how these values intersect with paleogenomic research approaches and outcomes allows researchers to adapt to the diverse needs embedded in aDNA research projects. To summarize these interconnecting themes, Figure 1 depicts a framework that centers the various stakeholder communities and addresses four main ethical priorities: upholding community research capacity, supporting data sovereignty, co-interpreting and contextualizing research findings, and communicating research implications. Stakeholders and priorities are bridged by two questions (connected by arrows in the figure). These questions guide researchers in thinking about how to enact respect and care for community stakeholders, given anticipated scientific and social research outcomes. This figure illustrates the interconnected relationship of stakeholder communities and ethical priorities. Additionally, it provides probing questions that assist researchers with cultivating an ethical ethos in paleogenomic research.

Guidance for ethical approaches in paleogenomic research will ultimately continue to develop in response to old paradigms and new challenges. In the epigraph at the beginning of this review, Kaestle & Horsburgh (64) stated that “we must regulate ourselves” amid the spectrum of guidance and perspectives (p. 109). The past 20 years of conversations concerning ethics in aDNA have followed suit, with varying opinions regarding best practices for the field. However, we must ask ourselves, Have we been successful? In a recent commentary, Tsosie et al. (111) wrote that the field should be skeptical of “guidelines that do not meet or exceed more stringent ethical standards to minimize harm and maximize benefits to communities” (p. 37). Ultimately, the rights and needs of descendants and affiliated communities who are affected by aDNA research should be at the center of these conversations. As with research with living people, science must be held accountable to the communities with which they work. We challenge the field to more deeply engage with understanding dynamic consent and to align research needs with the priorities of stakeholder communities in paleogenomic research. We all have a responsibility to be ethical ancestors ourselves and reflect on how we would want future communities to think carefully about these questions.

## CONCLUSION

In this review, we have summarized the current literature on ethical guidance for aDNA research concerning Ancestors. These guiding principles serve to assist researchers with designing or directing aDNA research that recognizes legal, historical, social, cultural, and ethical issues for stakeholder communities. Current guidance reveals varying approaches and frameworks for ethics in paleogenomics, ranging from prioritizing the needs of stakeholder communities to those of the scientific community. The variety of temporal, geographic, and cultural contexts in aDNA research results in a diverse set of needs for each project undertaken. Guidance that centers community stakeholders might be the most effective means to obtain authentic support given this diversity, as these approaches can design engagement methods that are flexible enough to include a wide range of values (10, 125). While guidance focused on the needs of the scientific community is important to ensure scientific reproducibility while minimizing physical harm to samples from Ancestors, deeper considerations about how these guidelines will be enforced and who will be held accountable are needed as the field moves forward.

## Figures and Tables

**Table 1:** Examples of early guidance on ethics in ancient DNA research (pre-2009)

Year	Reference	Recommendations, principles, or guiding questions
2002	Kaestle & Horsburgh (64)	1. Does the application of the method address an anthropological question? 2. Are there nondestructive methods that can be used to achieve the result? 3. Do the conditions of the remains or other material suggest that ancient DNA is more likely to be present than not? 4. How will different stakeholders view the destruction of the remains in question? 5. What are the ethical, legal, and social implications of possible study results, if any, for living groups? 6. Has a reasonable attempt been made to define and receive informed consent from different stakeholders?
2003	Sealy (97)	1. Respect for the mortal remains of the dead shall be accorded to all, irrespective of origin, race, religion, nationality, custom, and tradition. 2. Respect for the wishes of the dead concerning disposition shall be accorded whenever possible, reasonable, and lawful, when they are known or can be reasonably inferred. 3. Respect for the wishes of the local community and of relatives or guardians of the dead shall be accorded whenever possible, reasonable, and lawful. 4. Respect for the scientific research value of skeletal, mummified, and other human remains (including fossil hominids) shall be accorded when such value is demonstrated to exist. 5. Agreement on the disposition of fossil, skeletal, mummified, and other remains shall be reached by negotiation on the basis of mutual respect for the legitimate concerns of communities for the proper disposition of their Ancestors, as well as the legitimate concerns of science and education. 6. The express recognition that the concerns of various ethnic groups, as well as those of science, are legitimate and are to be respected will permit acceptable agreements to be reached and honored.
2008	Hublin et al. (59)	1. The scientific question addressed should be important enough to justify invasive sampling of hominid remains and should not be answerable by any other means. 2. If abundant and/or less unique animal fossils are present at a site, the invasive techniques should be shown to be successfully applied to such remains before hominid fossils are sampled. Whenever possible, minimally destructive tests able to predict whether the planned analysis can be successful should be performed on the hominid specimen prior to the sampling. 3. The scientist suggesting invasive sampling must demonstrate a relevant publication record. The more unique a specimen is, the higher the standards should be. This applies in particular to type specimens. Envisioned protocol, equipment, long-term funding, and archival resources should all be considered in relation to the project suggested. A detailed application should be presented to the curators. If the institution curating the remains does not have adequate in-house expertise to judge the track record of the applicant and the research proposal, the application should be sent by the curators to external reviewers. 4. Both negative and positive results should be reported back to curators and published in papers and/or online databases. 5. Redundant (duplicate) sampling should be done only when scientifically absolutely necessary. Whenever possible, sampling should be minimized by performing different types of analyses on the same sample. Regarding specimens that yielded negative results, requests for renewed sampling should be granted only when new technologies or new sampling procedures are available.

We defined early guidance to encompass publications released before 2009, which corresponds to the period prior to the release of the draft Neanderthal genome sequence and the resulting proliferation of ancient genomic studies (53). Some guidelines have been abbreviated for length.

**Table 2:** Examples of recent guidance on ethics in ancient DNA research (post-2009)

Year	Reference	Recommendations, principles, or guiding questions
2017	Claw et al. (29)	1. Museums and federal agencies tasked with protecting Native American Ancestors should make determinations of culturally unidentifiable remains in consultation with tribal experts, respectfully granting equal weight to tribal ways of knowing and histories when evaluating cultural affiliation. 2. Museums and entities that manage archaeological collections should support the formation of intermuseum meetings and coordination to share best practices in tribal consultation. 3. All studies involving Native American Ancestors should consult with tribes, including not only those deemed to be culturally affiliated but also those with historical and geographical ties to the area. 4. Scientific journals and granting bodies should ensure that ethical research practices are followed before publication and throughout the research process by requiring evidence of meaningful tribal consultation, especially when Native American Ancestors are involved.
2018	Bardill et al. (10)	1. In the absence of known descendant or culturally affiliated communities, which Indigenous peoples tied to land where Ancestors were buried will be consulted? 2. Who is the appropriate community body (e.g., tribal council, tribal institutional review board, or Elders) or representative (e.g., tribal president or historic preservation officer) to initiate discussions about paleogenomic analyses? 3. What are potential ethical pitfalls of this research or harms that could affect the community? What cultural concerns of the community, such as destruction of Ancestral remains, need to be considered? 4. How will the community benefit from the paleogenomic research? 5. How will the community provide input on study design and interpretation of results? How frequently does the community wish to be contacted during the project? 6. When community members participate directly in the project (e.g., as advisers or laboratory technicians), will they coauthor research publications and presentations? How do communities and individuals wish to be recognized in research products? 7. What happens after the project ends? Who will have access to the data generated? How will remaining samples from Ancestors be handled, stored, returned, or reburied?
	Prendergast & Sawchuk (91)	1. *Getting started: developing a research project* 1.1. Researchers must identify and listen to key stakeholders, while being specific about project goals and explicit about proposed sample destruction. 1.2. Country- and institution-specific research and export requirements must be determined, and budgets must account not only for sampling but also for sample return and continued engagement with collaborating institutions. 1.3. Archaeologists should not feel bound to any single laboratory or researcher, but rather choose appropriate laboratories and techniques based on research questions. 1.4. It is imperative to avoid a “sample first, ask questions later” approach. An ethical approach identifies specific sites, contexts, and individuals required to meet defined scientific goals. 1.5. It may be helpful to contact the excavators of targeted skeletons to obtain critical contextual or preservation information unavailable in publications or accession registers. 1.6. Sampling teams must include appropriate specialists, ideally a bioarchaeologist or osteologist and somebody trained in aDNA sampling techniques (98). 1.7. All parties should agree to terms of collaboration, ideally through a memorandum of understanding between or among institutions rather than individuals. 2. *Selection and documentation of tissue samples from collections* 2.1. Sampling teams must establish protocols to minimize contamination at all stages of collection and to fully document sampling procedures for the benefit of curating institutions and future researchers. 2.2. Researchers should minimize impacts on future bioarchaeological research by choosing samples that are less informative about the individual’s age, sex, disease, or life history. 2.3. No more than two tissue samples per individual should be collected without consultation with curators and reasonable justification tied to research questions.
	Prendergast & Sawchuk (91)	3. *Research does not end in the laboratory: following through on collaborations* 3.1. Researchers must adhere to plans for sample return and archiving within the minimum time necessary to ensure quality research. 3.2. When samples are returned, electronic and paper documentation should be updated accordingly. 3.3. Institutional collaborators should be involved in the interpretation and copublication of results, which may require in-person follow-up meetings. 3.4. Researchers should reach beyond the scientific community to communicate findings to public audiences—for example, in pamphlet or poster format or through local presentations. 3.5. Researchers should strive to maintain long-term ties with collaborators and colleagues and to build capacities by developing new research projects, mentoring, and cowriting communications.
2019	Austin et al. (8)	1. Researchers should “consult with descendant communities at the earliest stage of project design” (p. 1472). 2. “Decisions about destructive sampling are based, in part, on the likelihood that the proposed analytical methods will yield the intended results and gain the most possible information from the sampled collections” (p. 1473). 3. Researchers should provide for “accessibility of raw data to ensure complete replicability of research and stable, open access to data deriving from collections” (p. 1474).
	Sirak & Sedig (99)	1. It is important to identify the research questions that will be addressed with paleogenomic data to determine the number of samples that are needed to meaningfully contribute to the resolution of these questions. 2. Researchers should be realistic about the likelihood of analytical success and consider how results will be disseminated. 3. Researchers should fully assess the chances of generating robust data from the petrous bone as opposed to other skeletal elements. 4. Raw sequencing data should be deposited in a publicly accessible repository, and all protocols used should be fully reported.
2020	Wagner et al. (American Society of Human Genetics) (125)	1. Researchers should formally consult with communities. 2. Researchers should address cultural and ethical considerations. 3. Researchers should engage communities and support capacity building. 4. Researchers should develop plans to report results and manage data. 5. Researchers should develop plans for long-term responsibility and stewardship.
2021	Society for American Archaeology (102)	1. Working with human remains is a privilege, not a right. 2. Human remains should be treated with dignity and respect. 3. Archaeologists should consult, collaborate, and obtain consent when working with human remains. 4. It is the archaeologists’ responsibility to understand and comply with the applicable law. 5. Archaeologists should follow best practices and uphold the highest ethical standards when working with human remains.
	Alpaslan-Roodenberg et al. (1)	1. Researchers must ensure that all regulations were followed in the places where they work and from which the human remains derived. 2. Researchers must prepare a detailed plan prior to beginning any study. 3. Researchers must minimize damage to human remains. 4. Researchers must ensure that data are made available following publication to allow critical reexamination of scientific findings. 5. Researchers must engage with other stakeholders from the beginning of a study and ensure respect and sensitivity to stakeholder perspectives.

We defined recent guidance to encompass publications released after 2009, which corresponds to the period after the release of the draft Neanderthal genome sequence and the resulting proliferation of ancient genomic studies (53). Some guidelines have been abbreviated for length.

## HAVASUPAI TRIBE VERSUS THE ARIZONA BOARD OF REGENTS

Researchers at Arizona State University initially consented members of the Havasupai Tribe to study genetic associations related to increasing type 2 diabetes rates in their community (46). However, the tribe later learned that their members’ DNA was used without additional consent to conduct potentially stigmatizing genetic studies. In addition to studying mental conditions such as schizophrenia, researchers published population migration narratives that were culturally inconsistent with the tribe’s origin stories, using terms such as inbreeding to inaccurately depict population reductions due to colonial factors. In 2004, after discovering the unconsented use of their data, the Havasupai Tribe filed a lawsuit against the Arizona Board of Regents, which serves as the governing body for the university, for violations of informed consent. The lawsuit was settled in 2010 with monetary compensation and return of DNA samples to the community (35). The repercussions included increased caution by tribes about participating in research (2) and hesitance by researchers to engage Indigenous peoples in genetic research, raising important questions about justice and inclusion of underrepresented peoples in research (46). This seminal lawsuit also reflects the pitfalls of broad consent and demonstrates the importance of continually engaging Indigenous peoples to avoid research harms (48).

## THE GENOGRAPHIC PROJECT

In 2005, the National Geographic Society launched the Genographic Project, a genetic anthropological study to map ancient migratory patterns using DNA and create an open source research database (13, 128). The Indigenous Peoples Council on Biocolonialism immediately lambasted the project for its scientific objectivism of Indigenous peoples, destruction and desecration of Ancestors, and sustained bioethical and cultural concerns that recall similar issues raised by the government-funded Human Genome Diversity Project (57). Additionally, the project’s aims to scientifically reify African diaspora hypotheses using problematic phrases such as “we are all African,” “genetic science can end racism,” and “Indigenous peoples are vanishing” perpetuated controversial racial and colonial notions that pervade genetic research and contribute to erasures of Indigenous peoples’ histories, knowledges, and sovereignties (105). The privately funded project fueled ongoing concerns that collectivizing Indigenous peoples’ DNA in open databases would lead to biocommercialism and exploitation of their genomes for little or no benefit, but with disproportionate risks, to Indigenous communities. The aftermath of the project has further contributed to a divide between Indigenous peoples and scientists (55, 62). Hence, paleogenomicists should consider the importance of appropriate consultation and regard for risks to Indigenous peoples and pursue anticolonial models of engagement.

## IDENTIFYING RELATIVES OF TATANKA IYOTAKE (SITTING BULL)

The study examining genetic evidence of relatedness between Lakota Sioux leader Tatanka Iyotake (Sitting Bull) and great-grandson Ernie LaPointe highlights potential ethical concerns related to consent and engagement in paleogenomic research (78). Researchers received individual consent from participants who contributed DNA samples but failed to engage and diligently consult with affiliated tribal governments prior to initiating the study and communicating results. The research team sent one email to the Standing Rock Sioux tribal chair during the height of the coronavirus disease 2019 (COVID-19) pandemic just prior to publication; this constitutes neither engagement nor tribal consultation, only attempted contact. While individual consent is absolutely essential, participation in genetic research can have consequences for the broader affiliated community, such as tribes. Thus, multiple levels of engagement are necessary (110). Inadequate engagement with stakeholder communities is problematic because it exacerbates the cycle of extraction, exploitation, and distrust, undermining Indigenous sovereignty. Additionally, as the family’s oral history was previously verified by the Smithsonian (124), the overall utility of the genetic study is brought into question. This genetic study highlights long-standing concerns about using DNA to substantiate identity claims (106). Furthermore, it is potentially harmful to use Indigenous DNA for proof-of-concept methodological research, particularly from a revered Indigenous Ancestor.

## References

[1] Alpaslan-Roodenberg S, Anthony D, Babiker H, Bánffy E, Booth T, 2021. Ethics of DNA research on human remains: five globally applicable guidelines. Nature 599:41–46 34671160 10.1038/s41586-021-04008-x PMC7612683

[2] Am. J. Med. Genet. 2010. After Havasupai litigation, Native Americans wary of genetic research. Am. J. Med. Genet. A 152A:ix 10.1002/ajmg.a.33592 20583166

[3] Anagnostou P, Capocasa M, Milia N, Sanna E, Battaggia C, 2015. When data sharing gets close to 100%: what human paleogenetics can teach the open science movement. PLOS ONE 10:e0121409 25799293 10.1371/journal.pone.0121409 PMC4370607

[4] Arbour L, Cook D. 2006. DNA on loan: issues to consider when carrying out genetic research with aboriginal families and communities. J. Commun. Genet 9:153–60 10.1159/000092651 16741344

[5] Atalay S. 2006. Indigenous archaeology as decolonizing practice. Am. Indian Q 30:280–310

[6] Atalay S. 2007. Global application of Indigenous archaeology: community based participatory research in Turkey. Archaeologies 3:249–70

[7] Atalay S. 2012. Community-Based Archaeology: Research with, by, and for Indigenous and Local Communities. Berkeley: Univ. Calif. Press

[8] Austin RM, Sholts SB, Williams L, Kistler L, Hofman CA. 2019. Opinion: to curate the molecular past, museums need a carefully considered set of best practices. PNAS 116:1471–74 30696775 10.1073/pnas.1822038116 PMC6358678

[9] Bader AC, Carbaugh AE, Bardill J, Malhi RS, Petzelt B, Mitchell J. 2020. Building relationships to shift accountability: doing paleogenomic research with Indigenous nations and Ancestors. In Working with and for Ancestors: Collaboration in the Care and Study of Ancestral Remains, ed. Meloche CH, Spake L, Nichols KL, pp. 166–77. NewYork: Routledge

[10] Bardill J, Bader AC, Garrison NA, Bolnick DA, Raff JA, 2018. Advancing the ethics of paleogenomics. Science 360:384–85 29700256 10.1126/science.aaq1131 PMC6150602

[11] Battle-Baptiste W. 2017. Black Feminist Archaeology. New York: Routledge

[12] Beauchamp TL. 2011. Informed consent: its history, meaning, and present challenges. Camb. Q. Healthc. Ethics 20:515–23 21843382 10.1017/S0963180111000259

[13] Behar DM, Rosset S, Blue-Smith J, Balanovsky O, Tzur S, 2007. The Genographic Project public participation mitochondrial DNA database. PLOS Genet. 3:e104 17604454 10.1371/journal.pgen.0030104 PMC1904368

[14] Benn Torres J. 2021. Who does our research serve? Paper presented at the Ethical Futures for Curation, Research, and Training in Biological Anthropology Conference, Smithsonian Institution, Washington, DC, Nov. 14–17

[15] Bhambra GK, Holmwood J. 2021. Colonialism and Modern Social Theory. Cambridge, UK: Polity

[16] Bolnick DA, Bonine HM, Mata-Míguez J, Kemp BM, Snow MH, LeBlanc SA. 2012. Nondestructive sampling of human skeletal remains yields ancient nuclear and mitochondrial DNA. Am. J. Phys. Anthropol 147:293–300 22183740 10.1002/ajpa.21647

[17] Boulton G, Rawlins M, Vallance P, Walport M. 2011. Science as a public enterprise: the case for open data. Lancet N. Am. Ed 377:1633–35 10.1016/S0140-6736(11)60647-8 21571134

[18] Brandt AM. 1978. Racism and research: the case of the Tuskegee Syphilis Study. Hastings Cent. Rep 8:21–29 721302

[19] Bruning SB. 2006. Complex legal legacies: the Native American Graves Protection and Repatriation Act, scientific study, and Kennewick Man. Am. Antiq 71:501–21

[20] Budin-Ljøsne I, Teare HJA, Kaye J, Beck S, Bentzen HB, 2017. Dynamic consent: a potential solution to some of the challenges of modern biomedical research. BMC Med. Ethics 18:4 28122615 10.1186/s12910-016-0162-9 PMC5264333

[21] Callaway E. 2017. Stop hoarding ancient bones, plead archaeologists. Nature, Aug. 11. 10.1038/nature.2017.22445

[22] Campbell EG, Louis KS, Blumenthal D. 1998. Looking a gift horse in the mouth: corporate gifts supporting life sciences research. JAMA 279:995–99 9533497 10.1001/jama.279.13.995

[23] Carroll SR, Garba I, Figueroa-Rodríguez OL, Holbrook J, Lovett R, 2020. The CARE principles for Indigenous data governance. Data Sci. J 19:43

[24] Cassell EJ. 2000. The principles of the Belmont report revisited: How have respect for persons, beneficence, and justice been applied to clinical medicine? Hastings Cent. Rep 30:12–21 10971887

[25] Chatters JC, Kennett DJ, Asmerom Y, Kemp BM, Polyak V, 2014. Late Pleistocene human skeleton and mtDNA link Paleoamericans and modern Native Americans. Science 344:750–54 24833392 10.1126/science.1252619

[26] Childress JF, Faden RR, Gaare RD, Gostin LO, Kahn J, 2002. Public health ethics: mapping the terrain. J. Law Med. Ethics 30:170–78 12066595 10.1111/j.1748-720x.2002.tb00384.x

[27] Citron DK, Solove DJ. 2022. Privacy harms. Boston Univ. Law Rev. 102:793–863

[28] Claw KG, Anderson MZ, Begay RL, Tsosie KS, Fox K, 2018. A framework for enhancing ethical genomic research with Indigenous communities. Nat. Commun 9:2957 30054469 10.1038/s41467-018-05188-3 PMC6063854

[29] Claw KG, Lippert D, Bardill J, Cordova A, Fox K, 2017. Chaco Canyon dig unearths ethical concerns. Hum. Biol 89:177–80 29745246 10.13110/humanbiology.89.3.01 PMC5951383

[30] Colwell-Chanthaphonh C. 2012. Archaeology and Indigenous collaboration. In Archaeological Theory Today, ed. Hodder I, pp. 267–91. Cambridge, UK: Polity. 2nd ed.

[31] Constantin A. 2018. Human subject research: international and regional human rights standards. Health Hum. Rights 20:137–48 30568408 PMC6293356

[32] Cortez AD, Bolnick DA, Nicholas G, Bardill J, Colwell C. 2021. An ethical crisis in ancient DNA research: insights from the Chaco Canyon controversy as a case study. J. Soc. Archaeol 21:157–78

[33] Counc. Int. Organ. Med. Sci. 2016. International Ethical Guidelines for Biomedical Research Involving Human Subjects. Geneva: Counc. Int. Organ. Med. Sci. 4th ed. https://cioms.ch/wp-content/uploads/2017/01/WEB-CIOMS-EthicalGuidelines.pdf

[34] Cramer M. 2021. DNA confirms Sitting Bull was South Dakota man’s great-grandfather. New York Times, Oct. 29. https://www.nytimes.com/2021/10/29/us/sitting-bull-dna-great-grandson.html

[35] Dalton R. 2004. When two tribes go to war. Nature 430:500–2 15282577 10.1038/430500a

[36] DeWitte SN. 2015. Bioarchaeology and the ethics of research using human skeletal remains. Hist. Compass 13:10–19

[37] Dunnavant J, Justinvil D, Colwell C. 2021. Craft an African American Graves Protection and Repatriation Act. Nature 593:337–40 34012089 10.1038/d41586-021-01320-4

[38] Einav S, Ranzani OT. 2020. Focus on better care and ethics: Are medical ethics lagging behind the development of new medical technologies? Intensive Care Med. 46:1611–13 32462323 10.1007/s00134-020-06112-4 PMC7251219

[39] Fine-Dare KS. 2002. Grave Injustice: The American Indian Repatriation Movement and NAGPRA. Lincoln: Univ. Neb. Press

[40] Fleskes RE, Ofunniyin AA, Gilmore JK, Poplin E, Abel SM, 2021. Ancestry, health, and lived experiences of enslaved Africans in 18th century Charleston: an osteobiographical analysis. Am. J. Phys. Anthropol 175:3–24 10.1002/ajpa.24149 33022107

[41] Forssén A, Meland E, Hetlevik I, Strand R. 2011. Rethinking scientific responsibility. J. Med. Ethics 37:299–302 21266389 10.1136/jme.2010.038828 PMC3088477

[42] Fox K. 2020. The illusion of inclusion—the “All of Us” research program and Indigenous peoples’ DNA. N. Engl. J. Med 383:411–13 32726527 10.1056/NEJMp1915987

[43] Fox K, Hawks J. 2019. Use ancient remains more wisely. Nature 572:581–83 31462783 10.1038/d41586-019-02516-5

[44] Franklin M. 2001. A Black feminist-inspired archaeology? J. Soc. Archaeol 1:108–25

[45] Funtowicz SO, Ravetz JR. 1993. Science for the post-normal age. Futures 25:739–55

[46] Garrison NA. 2013. Genomic justice for Native Americans: impact of the Havasupai Case on genetic research. Sci. Technol. Hum. Values 38:201–23 10.1177/0162243912470009 PMC5310710 28216801

[47] Garrison NA, Carroll SR, Hudson M. 2020. Entwined processes: rescripting consent and strengthening governance in genomics research with Indigenous communities. J. Law Med. Ethics 48:218–20 32342771 10.1177/1073110520917020

[48] Garrison NA, Cho MK. 2013. Awareness and acceptable practices: IRB and researcher reflections on the Havasupai lawsuit. AJOB Prim. Res 4:55–63 24089655 10.1080/21507716.2013.770104 PMC3786163

[49] Garrison NA, Hudson M, Ballantyne LL, Garba I, Martinez A, 2019. Genomic research through an Indigenous lens: understanding the expectations. Annu. Rev. Genom. Hum. Genet 20:495–517 10.1146/annurev-genom-083118-015434 30892943

[50] Gasser U, Ienca M, Scheibner J, Sleigh J, Vayena E. 2020. Digital tools against COVID-19: taxonomy, ethical challenges, and navigation aid. Lancet Digit. Health 2:e425–34 32835200 10.1016/S2589-7500(20)30137-0 PMC7324107

[51] Gibbon VE. 2020. African ancient DNA research requires robust ethics and permission protocols. Nat. Rev. Genet 21:645–47 32939074 10.1038/s41576-020-00285-w

[52] Gibbons A. 2016. Ancient DNA divide. Science 352:1384–87 27313020 10.1126/science.352.6292.1384

[53] Green RE, Krause J, Briggs AW, Maricic T, Stenzel U, 2010. A draft sequence of the Neandertal genome. Science 328:710–22 20448178 10.1126/science.1188021 PMC5100745

[54] Haraway D. 1988. Situated knowledges: the science question in feminism and the privilege of partial perspective. Fem. Stud 14:575–99

[55] Harmon A. 2006. DNA gatherers hit a snag: The tribes don’t trust them. New York Times, Dec. 10, pp. A1, A38. https://www.nytimes.com/2006/12/10/us/10dna.html 17167865

[56] Harney É, Cheronet O, Fernandes DM, Sirak K, Mah M, .2021.A minimally destructive protocol for DNA extraction from ancient teeth. Genome Res. 31:472–83 33579752 10.1101/gr.267534.120 PMC7919446

[57] Harry D, Kanehe LM, Pennisi E, Greely H. 2006. Genetic research: collecting blood to preserve culture. Cultural Survival Quarterly Magazine 29(4). https://www.culturalsurvival.org/publications/cultural-survival-quarterly/genetic-research-collecting-blood-preserve-culture

[58] Havasupai Tribe of the Havasupai Reservation v. Arizona Board of Regents, Az. Ct. App. 1 CA-CV 07-0454, 07–0801 (2008)

[59] Hublin J-J, Pääbo S, Derevianko AP, Doronichev VB, Golovanova LV, 2008. Suggested guidelines for invasive sampling of hominid remains. J. Hum. Evol 55:756–57 18721998 10.1016/j.jhevol.2008.04.010

[60] Hudson M, Garrison NA, Sterling R, Caron NR, Fox K, . 2020. Rights, interests and expectations: Indigenous perspectives on unrestricted access to genomic data. Nat. Rev. Genet 21:377–84 32251390 10.1038/s41576-020-0228-x

[61] Israel BA, Schulz AJ, Parker EA, Becker AB. 1998. Review of community-based research: assessing partnership approaches to improve public health. Annu. Rev. Public Health 19:173–202 9611617 10.1146/annurev.publhealth.19.1.173

[62] Jacobs B, Roffenbender J, Collmann J, Cherry K, Bitsói LL, 2010. Bridging the divide between genomic science and indigenous peoples. J. Law Med. Ethics 38:684–96 20880250 10.1111/j.1748-720X.2010.00521.x

[63] Jones JH. 2008. The Tuskegee Syphilis Experiment. In The Oxford Textbook of Clinical Research Ethics, ed. Grady CC, Wendler DD, Emanuel EJ, Miller FG, Lie RK, Crouch RA, pp. 86–96. Oxford, UK: Oxford Univ. Press

[64] Kaestle FA, Horsburgh KA. 2002. Ancient DNA in anthropology: methods, applications, and ethics. Am. J. Phys. Anthropol 119(Suppl. 35):92–130 12653310 10.1002/ajpa.10179

[65] Kass NE. 2017. A journey in public health ethics. Perspect. Biol. Med 60:103–16 28890452 10.1353/pbm.2017.0022

[66] Kennett DJ, Plog S, George RJ, Culleton BJ, Watson AS, 2017. Archaeogenomic evidence reveals prehistoric matrilineal dynasty. Nat. Commun 8:14115 28221340 10.1038/ncomms14115 PMC5321759

[67] Kitchin R. 2013. Four critiques of open data initiatives. Programmable City. http://progcity.maynoothuniversity.ie/2013/11/four-critiques-of-open-data-initiatives

[68] Ledford H. 2014. Indirect costs: keeping the lights on. Nature 515:326–29 25409809 10.1038/515326a

[69] Lee LM. 2017. A bridge back to the future: public health ethics, bioethics, and environmental ethics. Am. J. Bioeth 17:5–12 28829266 10.1080/15265161.2017.1353164

[70] Lepore J. 2021. When Black history is unearthed, who gets to speak for the dead? New Yorker, Sept. 27. https://www.newyorker.com/magazine/2021/10/04/when-black-history-is-unearthed-who-gets-to-speak-for-the-dead

[71] Lindo J, Achilli A, Perego UA, Archer D, Valdiosera C, 2017. Ancient individuals from the North American Northwest Coast reveal 10,000 years of regional genetic continuity. PNAS 114:4093–98 28377518 10.1073/pnas.1620410114 PMC5402414

[72] Lynott MJ, Wylie A, eds. 1995. Ethics in American Archaeology: Challenges for the 1990s. Washington, DC: Soc. Am. Archaeol.

[73] Makarewicz C, Marom N, Bar-Oz G. 2017. Palaeobiology: ensure equal access to ancient DNA. Nature 548:158 10.1038/548158a 28796208

[74] Marchant GE, Allenby BR, Herkert JR. 2011. The Growing Gap Between Emerging Technologies and Legal-Ethical Oversight: The Pacing Problem. London: Springer

[75] Marciniak S, Perry GH. 2017. Harnessing ancient genomes to study the history of human adaptation. Nat. Rev. Genet 18:659–74 28890534 10.1038/nrg.2017.65

[76] Mariella P, Brown E, Carter M, Verri V. 2009. Tribally-driven participatory research: state of the practice and potential strategies for the future. J. Health Dispar. Res. Pract 3(2):41–58

[77] Mittelstadt BD, Floridi L. 2016. The ethics of big data: current and foreseeable issues in biomedical contexts. Sci. Eng. Ethics 22:303–41 26002496 10.1007/s11948-015-9652-2

[78] Moltke I, Korneliussen TS, Seguin-Orlando A, Moreno-Mayar JV, LaPointe E, 2021. Identifying a living great-grandson of the Lakota Sioux leader Tatanka Iyotake (Sitting Bull). Sci. Adv 7:eabh2013 34705496 10.1126/sciadv.abh2013 PMC8550246

[79] Mount Isa Mines Ltd v. Pusey, 125 C.L.R. 383 (1970) (H. Ct. Aust.)

[80] Mulligan CJ. 2006. Anthropological applications of ancient DNA: problems and prospects. Am. Antiq 71:365–80

[81] Mullings L, Torres JB, Fuentes A, Gravlee CC, Roberts D, Thayer Z. 2021. The biology of racism. Am. Anthropol 123:671–80

[82] Natl. Comm. Prot. Hum. Subj. Biomed. Behav. Res. 1978. The Belmont Report: ethical principles and guidelines for the protection of human subjects of research. Rep., Natl. Comm. Prot. Hum. Subj. Biomed. Behav. Res., Washington, DC

[83] Natl. Mus. Am. Indian. 2022. Repatriation. National Museum of the American Indian. https://americanindian.si.edu/explore/repatriation

[84] Natl. Park Serv. 1998. Management of ethnographic resources. In NPS-28: Cultural Resource Management Guideline. Washington, DC: Natl. Park Serv. https://www.nps.gov/parkhistory/online_books/nps28/28chap10.htm

[85] Natl. Park Serv. 2021. Frequently asked questions: Who is responsible for complying with NAGPRA? National Park Service. https://www.nps.gov/subjects/nagpra/frequently-asked-questions.htm

[86] Nicholas GP. 2014. Indigenous archaeology. In Oxford Bibliographies: Anthropology, ed. Jackson JL Jr. Oxford, UK: Oxford Univ. Press. 10.1093/OBO/9780199766567-0073

[87] Nordmann A, Rip A. 2009. Mind the gap revisited. Nat. Nanotechnol 4:273–74 19421202 10.1038/nnano.2009.26

[88] Nowotny H, Scott PB, Gibbons MT. 2013. Re-Thinking Science: Knowledge and the Public in an Age of Uncertainty. New York: Wiley

[89] Orlando L, Allaby R, Skoglund P, Der Sarkissian C, Stockhammer PW, 2021.Ancient DNA analysis. Nat. Rev. Methods Primers 1:14

[90] O’Rourke DH, Geoffrey Hayes M, Carlyle SW. 2000. Ancient DNA studies in physical anthropology. Annu. Rev. Anthropol 29:217–42

[91] Prendergast ME, Sawchuk E. 2018. Boots on the ground in Africa’s ancient DNA “revolution”: archaeological perspectives on ethics and best practices. Antiquity 92:803–15

[92] Prictor M, Huebner S, Teare HJA, Burchill L, Kaye J. 2020. Australian Aboriginal and Torres Strait Islander collections of genetic heritage: the legal, ethical and practical considerations of a dynamic consent approach to decision making. J. Law Med. Ethics 48:205–17 32342777 10.1177/1073110520917012

[93] Pyburn KA. 2011. Engaged archaeology: Whose community? Which public? In New Perspectives in Global Public Archaeology, ed. Okamura K, Matsuda A, pp. 29–41. New York: Springer

[94] Reynolds A. 2018. Ancient DNA labs. Google Maps. https://www.google.com/maps/d/u/0/viewer?mid=1qwXOKV5uoQntgBsxQrxS01YHpbs&ll=52.19802207086801%2C-5.195632878906151&z=5

[95] Romain PL. 2015. Conflicts of interest in research: looking out for number one means keeping the primary interest front and center. Curr. Rev. Musculoskelet. Med 8:122–27 25851417 10.1007/s12178-015-9270-2 PMC4596167

[96] Scarre G. 2006. Can archaeology harm the dead? In The Ethics of Archaeology: Philosophical Perspectives on Archaeological Practice, ed. Scarre G, Scarre C, pp. 181–98. Cambridge, UK: Cambridge Univ. Press

[97] Sealy J. 2003. Managing collections of human remains in South African museums and universities: ethical policy-making and scientific value. S. Afr. J. Sci 99:238–39

[98] Sirak KA, Fernandes DM, Cheronet O, Novak M, Gamarra B, .2017.A minimally-invasive method for sampling human petrous bones from the cranial base for ancient DNA analysis. BioTechniques 62:283–89 28625158 10.2144/000114558

[99] Sirak KA, Sedig JW. 2019. Balancing analytical goals and anthropological stewardship in the midst of the paleogenomics revolution. World Arch aeol. 51:560–73

[100] Skoglund P, Mathieson I. 2018.Ancient genomics of modern humans: the first decade. Annu. Rev. Genom. Hum. Genet 19:381–404 10.1146/annurev-genom-083117-021749 29709204

[101] Smith RWA, Non AL. 2022. Assessing the achievements and uncertain future of paleoepigenomics. Epigenomics 14:167–73 34850636 10.2217/epi-2021-0382

[102] Soc. Am. Archaeol. 2021. Statement concerning the treatment of human remains. Statement Doc., Soc. Am. Archaeol., Washington, DC. https://documents.saa.org/container/docs/default-source/doc-careerpractice/statement-concerning-the-treatment-of-human-remains.pdf

[103] Stumpf G. 2009. Native American Graves Protection & Repatriation Act. Rep., Bur. Land Manag, US Dep. Interior, Washington, DC. ttps://www.ntc.blm.gov/krc/uploads/646/GuidetoNAGPRA.pdf

[104] Swain GR, Burns KA, Etkind P. 2008. Preparedness: medical ethics versus public health ethics. J. Public Health Manag. Pract 14:354–57 18552646 10.1097/01.PHH.0000324563.87780.67

[105] TallBear K. 2007. Narratives of race and indigeneity in the Genographic Project. J. Law Med. Ethics 35:412–24 17714251 10.1111/j.1748-720X.2007.00164.x

[106] TallBear K. 2013. Native American DNA: Tribal Belonging and the False Promise of Genetic Science. Minneapolis: Univ. Minn. Press

[107] Tobler R, Rohrlach A, Soubrier J, Bover P, Llamas B, 2017. Aboriginal mitogenomes reveal 50,000 years of regionalism in Australia. Nature 544:180–84 28273067 10.1038/nature21416

[108] Torres JB, Kittles RA. 2007. The relationship between “race” and genetics in biomedical research. Curr. Hypertens. Rep 9:196–201 17519124 10.1007/s11906-007-0035-1

[109] Trials of War Criminals Before the Nuernberg Military Tribunals Under Control Council Law No. 10, Vol. II: The Medical Case. 1949. Washington, DC: US Gov. Print. Off.

[110] Trinidad SB, Blacksher E, Woodbury RB, Hopkins SE, Burke W, 2021. Precision medicine research with American Indian and Alaska Native communities: results of a deliberative engagement with tribal leaders. Genet. Med 24:622–30 34906504 10.1016/j.gim.2021.11.003 PMC9754657

[111] Tsosie KS, Bader AC, Fox K, Bolnick DA, Garrison NA, Smith RWA. 2021. Ancient-DNA researchers write their own rules. Nature 600:37 10.1038/d41586-021-03542-y 34848867

[112] Tsosie KS, Begay RL, Fox K, Garrison NA. 2020. Generations of genomes: advances in paleogenomics technology and engagement for Indigenous people of the Americas. Curr. Opin. Genet. Dev 62:91–96 32721847 10.1016/j.gde.2020.06.010 PMC7484015

[113] Tsosie KS, Claw KG, Garrison NA. 2021. Considering “respect for sovereignty” beyond the Belmont Report and the Common Rule: ethical and legal implications for American Indian and Alaska Native peoples. Am. J. Bioeth 21:27–30 10.1080/15265161.2021.1968068 34554064

[114] Tsosie KS, Fox K, Yracheta JM. 2021. Genomics data: the broken promise is to Indigenous people. Nature 591:529 10.1038/d41586-021-00758-w 33742179

[115] Tsosie KS, Yracheta JM, Kolopenuk J, Smith RWA. 2021. Indigenous data sovereignties and data sharing in biological anthropology. Am. J. Phys. Anthropol 174:183–86 33244756 10.1002/ajpa.24184

[116] Tuana N. 2007. Conceptualizing moral literacy. J. Educ. Adm. Hist 45:364–78

[117] Turner H. 2020. Cataloguing Culture: Legacies of Colonialism in Museum Documentation. Vancouver, Can.: UBC Press

[118] Turner TR, ed. 2012. Biological Anthropology and Ethics: From Repatriation to Genetic Identity. Albany: State Univ. N.Y. Press

[119] Ubelaker DH, Grant LG. 1989. Human skeletal remains: preservation or reburial? Am. J. Phys. Anthropol 32(Suppl. 10):249–87

[120] UN Comm. Econ. Soc. Cult. Rights. 2020. General comment No. 25 (2020) on science and economic, social and cultural rights (article 15 (1) (b), (2), (3) and (4) of the International Covenant on Economic, Social and Cultural Rights). Doc. E/C.12/GC/25, UN, New York. https://undocs.org/E/C.12/GC/25

[121] UN Gen. Assem. 1948. Universal Declaration of Human Rights. Resolut. 217A, UN, New York. https://www.un.org/en/about-us/universal-declaration-of-human-rights

[122] UN Gen. Assem. 2008. United Nations Declaration on the Rights of Indigenous Peoples. Decl. A/RES/61/295, UN, New York

[123] UN Off. High Comm. Hum. Rights. 1966. International Covenant on Economic, Social and Cultural Rights. Covenant Doc., UN, New York. https://www.ohchr.org/en/professionalinterest/pages/cescr.aspx

[124] Vlamis K. 2021. Sitting Bull’s great-grandson say she always knew his ancestry but some historians would say ‘you cannot trust the Natives’ oral history.’ Insider, Nov. 2. https://www.insider.com/sitting-bull-great-grandson-always-knew-grandfather-dna-confirmation-2021-10

[125] Wagner JK, Colwell C, Claw KG, Stone AC, Bolnick DA, 2020. Fostering responsible research on ancient DNA. Am. J. Hum. Genet 107:183–95 32763189 10.1016/j.ajhg.2020.06.017 PMC7413888

[126] Watkins J. 2000. Indigenous Archaeology: American Indian Values and Scientific Practice. Walnut Creek, CA: AltaMira

[127] Watkins J. 2005. Through wary eyes: Indigenous perspectives on archaeology. Annu. Rev. Anthropol 34:429–49

[128] Wells S. 2007. Deep Ancestry: Inside the Genographic Project. Washington, DC: Natl. Geogr. Books

[129] Wickenheiser RA. 2019. A crosswalk from medical bioethics to forensic bioethics. Forensic Sci. Int. Synergy 1:35–44 32411952 10.1016/j.fsisyn.2019.03.002 PMC7219182

[130] Wilkinson MD, Dumontier M, Aalbersberg IJJ, Appleton G, Axton M, 2016. The FAIR Guiding Principles for scientific data management and stewardship. Sci. Data 3:160018 26978244 10.1038/sdata.2016.18 PMC4792175

[131] World Med. Assoc. 2013. World Medical Association Declaration of Helsinki: ethical principles for medical research involving human subjects. JAMA 310:2191–94 24141714 10.1001/jama.2013.281053

[132] Wright JL, Wasef S, Heupink TH, Westaway MC, Rasmussen S, 2018. Ancient nuclear genomes enable repatriation of Indigenous human remains. Sci. Adv 4:eaau5064 30585290 10.1126/sciadv.aau5064 PMC6300400

